# Learning the Fastest RNA Folding Path Based on Reinforcement Learning and Monte Carlo Tree Search

**DOI:** 10.3390/molecules26154420

**Published:** 2021-07-22

**Authors:** Kangkun Mao, Yi Xiao

**Affiliations:** School of Physics and Key Laboratory of Molecular Biophysics of the Ministry of Education, Huazhong University of Science and Technology, Wuhan 430074, China; mkk@hust.edu.cn

**Keywords:** RNA secondary structure, folding pathways, deep reinforcement learning

## Abstract

RNA molecules participate in many important biological processes, and they need to fold into well-defined secondary and tertiary structures to realize their functions. Like the well-known protein folding problem, there is also an RNA folding problem. The folding problem includes two aspects: structure prediction and folding mechanism. Although the former has been widely studied, the latter is still not well understood. Here we present a deep reinforcement learning algorithms 2dRNA-Fold to study the fastest folding paths of RNA secondary structure. 2dRNA-Fold uses a neural network combined with Monte Carlo tree search to select residue pairing step by step according to a given RNA sequence until the final secondary structure is formed. We apply 2dRNA-Fold to several short RNA molecules and one longer RNA 1Y26 and find that their fastest folding paths show some interesting features. 2dRNA-Fold is further trained using a set of RNA molecules from the dataset bpRNA and is used to predict RNA secondary structure. Since in 2dRNA-Fold the scoring to determine next step is based on possible base pairings, the learned or predicted fastest folding path may not agree with the actual folding paths determined by free energy according to physical laws.

## 1. Introduction

More and more studies have shown that ribonucleic acid (RNA) participates in many important biological processes [1,2,3,4]. They need to fold into well-defined secondary and tertiary structures to perform their functions. Therefore, like the well-known “protein folding problem”, the “RNA folding problem” is a fundamental problem of RNA biology [5]. The “RNA folding problem” mainly includes two problems: RNA structure prediction and folding mechanism. The former deals with the determination of secondary and tertiary structures of an RNA directly from its sequences and has been widely studied [6,7,8,9,10,11,12,13,14,15]. The latter studies how an RNA molecule folds into its secondary and tertiary structures and is still not well understood although there have been some experimental/theoretical studies on RNA folding pathway [16,17], like the hairpin formation [18,19,20]. Here we shall focus on the problem of the RNA folding pathway in secondary structure formation.

Reinforcement learning is a computational method of understanding and automating goal-oriented learning and decision-making. The difference between it and other computational methods (deep learning) is that it emphasizes that the agent learns from direct interaction with the environment. Now reinforcement learning and deep learning are more and more combined together, referred as deep reinforcement learning methods. Deep reinforcement learning has now shown extraordinary results in many fields, such as AlphaGo [21] and AlphaGo Zero [22] for playing Go, AlphaStar for StarCraft game [23], OpenAI Five for the multiplayer video game Dota2 [24], and Pluribus for six-player no-limit Texas Hold’em [25]. The deep reinforcement learning has also begun to be used in the biological field, for example, in the design of RNA molecules [26].

From the perspective of exploring RNA folding path in secondary structure formation, here we propose an RNA folding path learning algorithm based on deep reinforcement learning, called 2dRNA-Fold. 2dRNA-Fold selects residue pairing step by step according to a given RNA sequence until the final secondary structure is formed. Then, we can find a folding path with the largest probability that can be considered as the fastest folding path of the RNA because of the special scoring function used.

## 2. Methods

**Figure 1:**
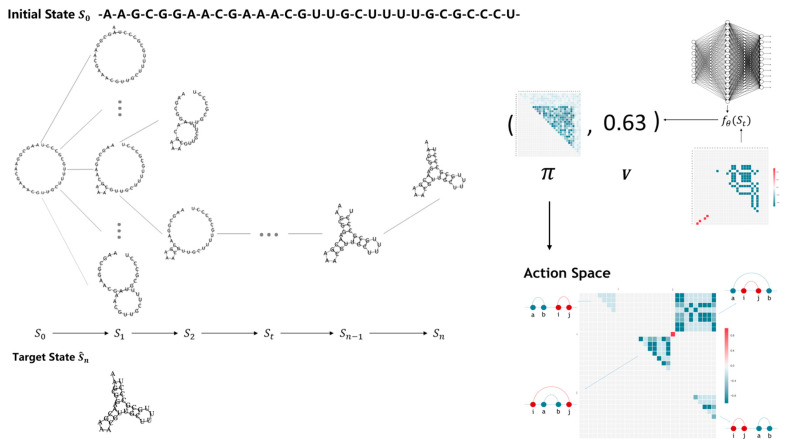
The flow chart of 2dRNA-Fold.

The flow chart of 2dRNA-Fold, a folding-path learning algorithm for RNA secondary structure based on deep reinforcement learning, is shown in Figure 1. In general, given a target RNA at first, its initial state $S_{0}$ is an open strand without any base pairing. The native RNA secondary structure is the target state ${\hat{S}}_{n}$. 2dRNA-Fold uses value function and policy function (both are neural networks), combining with Monte Carlo tree search, to select next base pair from the action space (this space consists of all the remaining possible pairing choices), and proceed to the next State $S_{t}$. The step-by-step selection of base pairing stops until the value function gives a stop signal by evaluating the current state. This ending state is the final state $S_{n}$, and this round of process ends. By comparing with the native secondary structure ${\hat{S}}_{n}$, the reward r is returned. 2dRNA-Fold learns episode by episode through these reward signals, and finally and stably goes to the native state. In this process, the base pairings selected at each step from the initial state to the native state form the folding path predicted by 2dRNA-Fold. We will explain the details in the following sections.

### 2.1. Reinforcement Learning

The main features of reinforcement learning are the agent and the environment. The environment is the world where the agent is in and interacts with. In each step of the interaction, the agent will see all or part of the state of the world, and then decide the action to be taken. The agent can also get a reward from the environment, which tells it the current state of the world. The goal of the agent is to maximize its cumulative return. Reinforcement learning is a way to maximize returns through learning [27].

In reinforcement learning, the optimization policy function can be updated according to the policy gradient. The policy gradient can be written as:

(1) $$g=E\left[\overset{\infty }{\underset{t=0}{\sum }}\Phi_{t}\nabla_{\theta }\log\pi_{\theta }\left(a_{t}|s_{t}\right)\right]$$

The gradient g is the derivation of expected reward, where $\pi_{\theta }\left(a_{t}|s_{t}\right)$ is Actor which represents the probability of taking action $a_{t}$ in the state $s_{t}$ and $\Phi_{t}$ is Critic indicating expected reward for situation $\left(s_{t},\;a_{t}\right)$, θ is the weights and biases of a neural network. This is a generalized Actor-Critic framework.

The reinforcement learning algorithm used by 2dRNA-Fold is the Advantage Actor-Critic (A2C) [28], and so the $\Phi_{t}$ function here is the advantage function $A^{\pi }\left(s_{t},\;a_{t}\right)$. It describes how well the policy π performing a specific action a in state s is better than choosing an action randomly. Mathematically, the advantage function is defined as:

(2) $$A^{\pi }\left(s,a\right)=Q^{\pi }\left(s,a\right)-V^{\pi }\left(s\right)$$

Here, $Q^{\pi }\left(s,a\right)$ is action-value function, which gives the expected return if you start in state *s*, take an action *a*, and then forever after acting according to the policy π. $V^{\pi }\left(s\right)$ is value function, which gives the expected return if you start in state *s* and always act according to the policy π. The advantage function $A^{\pi }\left(s,a\right)$ measures how much is taking a specific action a in state s better than randomly selecting an action according to π(·|s). If the advantage function is greater than zero, it means that the action is better than the average action (here “average” means *π* weighted sum over all actions). If the advantage function is less than zero, it means that the current action is not as good as the average action, which makes feedback with positives and negatives. Another superiority of advantage function is that the variance is small when training, because it uses the approximation method, but there will be some deviations in the calculated policy gradient. All in all, the advantage function makes the training process more stable than using the traditional trajectory return.

### 2.2. Monte Carlo Tree Search

Monte Carlo tree search (MCTS) [29,30] is a general term for a type of tree search algorithm. Compared with simply using the Monte Carlo algorithm to randomly sample in a huge search space, the Monte Carlo tree search algorithm expands the search space into a tree structure and performs a search on each tree node. Scoring can effectively prune and reduce unnecessary exploration. It combines the generality of random simulation and the accuracy of tree search and can solve some problems with huge search space. Monte Carlo tree searches include four steps, which are executed in sequence to form a loop.

(1) Selection

Selection is the most important step in Monte Carlo tree search. Start from the root node and go down its child nodes until the last leaf node. In the process of going down, there is a scoring function that will score each child node, so each time the child node with the highest score is selected to go down. This scoring algorithm generally uses the Upper Confidence Bounds to Trees (UCT) algorithm [31]. The scoring function strikes a balance between the exploration and utilization of each node, and its formula is as follows:

(3) $$\frac{W}{N}+c\sqrt{\frac{ln\;T}{N}}$$

where W represents how many times the node finally reach the state of success (e.g., win the game in chess or RNA folds to its native state), N represents the number of simulations of the node, and c is the exploration parameter. The larger the c is, the more likely it is to explore the nodes that have not been walked, Generally, it is set to $\sqrt{2}$. T represents the total number of simulations, equal to the sum of N for all nodes.

(2) Expansion

If the leaf node is the final state, then the tree search process ends. Otherwise, create a child node under the node and select one of the child nodes.

(3) Simulation

For fast simulation of the currently selected child node, random strategies can be used, and the final result can be obtained in a short time.

(4) Backpropagation

Using the simulation results of the current child node the information (W and N) of each passing node in the upward direction all the way to the root node is updated and finally the total number of simulations T is increased by one.

In 2dRNA-Fold, the Monte Carlo tree search algorithm is used when selecting the next base pair from the action space at each step. We did not use the standard Monte Carlo tree search algorithm, but some modifications. In the expansion step, we take the current RNA base pairing state as the root node, and its expanded child nodes are all possible base pairings in the current action space. Each of our nodes has three attributes, V, N and P, which represent the value of the node (given by the value function), the number of times the node has been passed and the probability of selecting this node (given by the policy π function), respectively. Therefore, the scoring function we used has changed accordingly, named as 2dRNA-Score:

(4) $$-V+cP\sqrt{\frac{N^{\prime }}{1+N}}$$

where the negative sign of V is because in our method V represents the error from the native state structure, which is estimated by the value function (neural network). Since the current state and native state are represented by a matrix of L×L, the real error is calculated as the sum of the difference between two matrixes. c is the exploration parameter, it strikes a balance between the exploration and utilization of each node. The larger the *c* is, the more likely it is to explore the nodes that have not been walked, we set it as 5 to encourage exploration. $N^{\prime }$ refers to the number of the parent nodes that have been passed, and 1+N is to avoid denominator being 0.

In the simulation step, we did not adopt a random strategy to select base pairings for simulation, which would take too long. Instead, we used the value function to directly predict the value V of the next state. In the backpropagation process, the value is returned to every node passed by. Another point worth noting is that after the state $S_{t}$ to $S_{t+1}$, the root node becomes $S_{t+1}$, but we will not delete the entire tree and start the Monte Carlo tree search from scratch. Instead, we choose $S_{t+1}$ as the root node and leave all the child nodes as same as the previous step, and then perform further Monte Carlo tree search. The above process can be seen in the left half of Figure 1. At each step, we will perform 2000 Monte Carlo tree searches to determine which base pair to choose in the next step.

### 2.3. The Fastest Folding Path Learning

**Figure 2:**
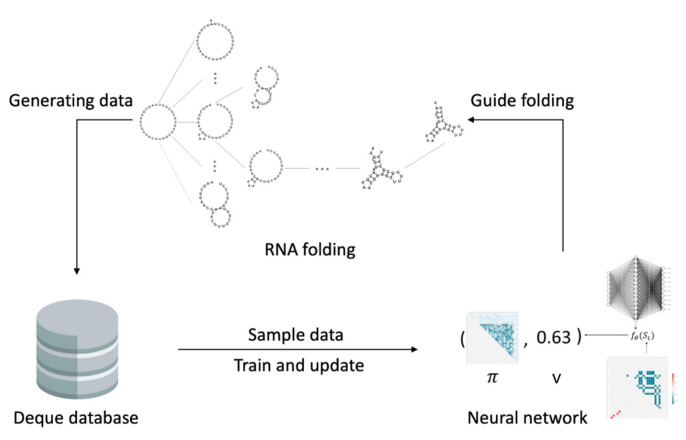
Diagram of the learning process of RNA molecular secondary structure folding path.

The diagram of 2dRNA-Fold predicts the folding path of a single RNA molecule as shown in Figure 2. The whole process starts from inputting the sequence of the target RNA, its initial state $S_{0}$ is an open chain without any base pairing, and then according to the policy π (the policy function here is learned by neural network) combined with Monte Carlo tree search algorithm, selects the next most likely base pairing from the action space. This is repeated until folding to the final state $S_{n}$. Here we use the value function (also learned by neural network) to score the current state to determine whether to stop. By comparing the final folding state $S_{n}$ with the native state ${\hat{S}}_{n}$, the error between the secondary structure is returned as a reward, and then this episode of folding simulation ends. All the simulated folding data of each episode are stored in the database, which has certain size and adopts a first-in-first-out queue. New data will squeeze out the oldest data, and then the algorithm randomly samples part of the data from the database after each episode to train and update policy function and value function. After a certain number of episodes of simulated folding and training, the policy function and value function will finally converge to a stable state. At this time, the most likely folding path can be obtained by statistical analyzing all the successful folding paths during the training process. Since in 2dRNA-Fold the scoring to determine next step is based on possible native base pairing, the learned most likely folding path can be considered as the fastest folding path.

#### 2.3.1. Input Data

The input data of the neural network in 2dRNA-Fold is a 18×L×L three-dimensional vector, where L is the length of the RNA sequence. The three-dimensional vector can be divided into three parts: the first part is used to encode the sequence information of the RNA, and the size is a matrix of 16×L×L. Because there are four types of RNA nucleotide bases (A, U, C, G), the number of the combinations of any two is 16, so we use a 16-layer L×L matrix to represent the sequence information. The second part uses a layer of L×L matrix to identify all current possible pairing positions. If the base pairing (i,j) can form a pairing, it is marked as 1, otherwise it is 0. The third part also uses a layer of L×L matrix to identify the base pairings that have been found. At the beginning, the matrix is all 0, because there is no pairing at this time, and the corresponding bases will be adjusted as the folding progresses, e.g., if base pair (i,j) is selected then it is set to 1. Finally, these three parts are stacked together to form the input 18×L×L matrix. It is worth noting that the data of the last two layers will dynamically change as the folding progresses, while the sequence coding information remains unchanged.

#### 2.3.2. Neural Network

**Figure 3:**
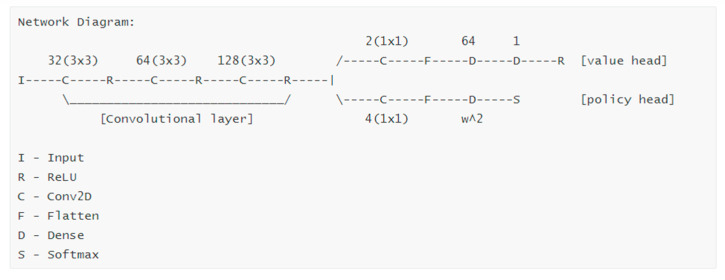
The neural network architecture of the policy function and value function.

The policy function and value function of 2dRNA-Fold use the same neural network, but there are two output heads to output the corresponding results respectively. The advantage of sharing a neural network is that it saves computational expenses and model expenses, training process is stable, and performance is better. Its network architecture is shown in Figure 3.

In the network architecture shown by Figure 3, a three-layer convolutional layer is shared in front of the network, the convolution channels are 32, 64, and 128, and the size of the convolution kernel is 3×3. Each convolutional layer is followed by a ReLU [32] activation function. Then there are two heads that represent the value function and the policy function. The value header outputs a scalar greater than zero, and the policy header outputs a vector of size $1\times L^{2}$ after flattening. Each value in this vector represents the pairing probability of each pair of bases. The value header uses the square error as the loss function, the policy header uses the cross entropy as the loss function, the optimizer both uses Adam [33], and the learning rate is set to 0.001.

#### 2.3.3. Action Space

**Figure 4:**
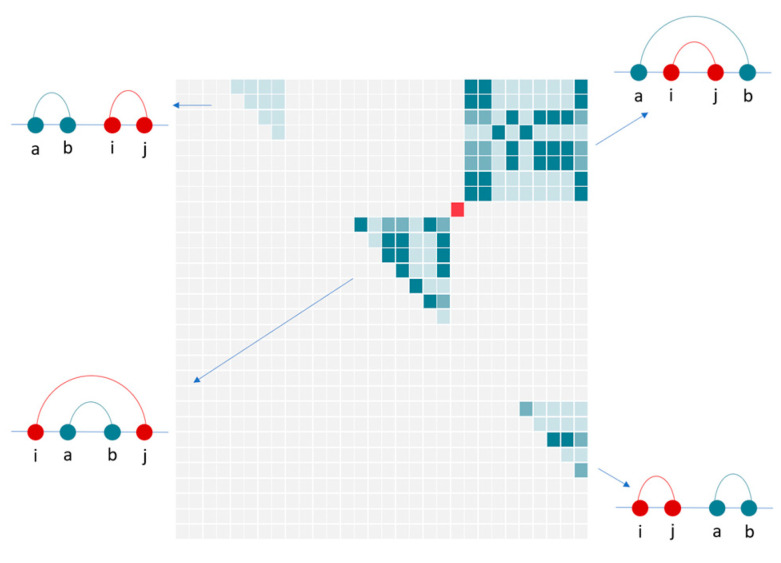
Select the action space left after base pairing (i,j). In the figure, each box represents one pair of residues, and sequence directions is from left to right and top to down. The red square represents the currently selected base pairing, noted as (i, j), the blue area represents the remaining base pairing area that can be formed, referred to as (a, b); the dark blue inside represents standard base pairing, and the light blue represents Wobble base pairing. The case of pseudoknots is not considered here.

We know that the base pairing in the secondary structure is not arbitrarily selected, but generally meets the following constraints: (1) A single base cannot form a pair with multiple bases; (2) At least three bases apart along sequence between bases that form a pair. Therefore, after filtering according to the above constraints, we can get a set that can form base pairings, which is called action space in reinforcement learning. As shown in Figure 4, when the base pairing (i,j) is selected (marked in red in the figure), the remaining action space is divided into four parts, and they represent four different possible situations: −(a,b)−(i,j)−, −(i,j)−(a,b)−, −(a,−(i,j)−,b)−, −(i,−(a,b)−,j)−. It is noted that pseudoknots are not considered here. We can find that every time a base pair is selected from the action space, the remaining possibilities are greatly reduced.

#### 2.3.4. Reinforcement Learning

For a given target RNA sequence, we will perform 1000 episodes of folding simulation. In each episode, each step of the base-pairing selecting process will perform 2000 Monte Carlo tree searches, and finally select the next possible base pair with the highest score, update its own state and action space, and then use the current state node as the root node to perform the next Monte Carlo tree search. This iterates until it reaches the final state judged by the value function, and then the episode ends. As mentioned in the input section above, the third part of the input data describes the current state, represented by a matrix of 1×L×L, so we use folded final state to compare with the native state, and calculate the sum of the squared errors between two states as the reward r for the episode as a whole. The reward is positive, and the larger it is, the greater the error is, so the direction of optimization of reinforcement learning is minimizing the overall reward. In the base pairing process of the ith step of each episode, a set of data will be generated, including the value of the current state $v_{i}$, the probability of the action space $p_{i}$, which size is L×L, denoted as $\left(v_{i},\;p_{i}\right)$. This set of data is calculated by the neural network and then corrected by Monte Carlo tree search. When this episode is over, the reward r is added to the data of each step, and finally $\left(v_{i},\;p_{i},\;r\right)$ is obtained. These data will then be added to a queue-based database with a size of 100,000, and new data added after this size will occupy the earliest stored data. Then, randomly sampling 2024 sets of data from it to train the value/policy neural network. After training, perform the next episode of folding simulation process on the new model, and continuously loop the above process until 1000 episodes.

#### 2.3.5. Gym Environment

Unlike machine learning or deep learning, which only needs to provide training data to train the model, the training of reinforcement learning algorithms generally requires an interactive environment for learning. OpenAI Gym [34] is such a module, which provides many excellent simulation environments, so that our reinforcement learning algorithm can be easily trained in it. Therefore, based on the Gym module, we built a set of reinforcement learning simulation environment for RNA structure prediction, including RNA secondary structure prediction and tertiary structure prediction. We call this Gym environment RNAWorld. Two gym environment classes are implemented in the RNAWorld environment: RNAWorld2D and RNAWorld3D, which correspond to the RNA secondary structure prediction environment and the RNA tertiary structure prediction environment, respectively.

In the RNAWorld2D environment, the secondary structure of the native RNA only contains standard base pairings A-U and C-G and wobble base pairing G-U, but it allows pseudoknots. Here we also provide two modes to choose from, the one with pseudoknots and the one without pseudoknots. Depending on the mode selection, the action space (the remaining optional base pairing) will be different. At the beginning of each episode, it will initialize the RNA secondary structure to an open strand, and the action space is all possible base pairings (A-U, G-C, G-U). The current RNA state is an L×L matrix, where 1 means pairing, and 0 means no pairing. As each episode progresses, we select a possible base pair from the current action space and pass it to the step() function which updates the RNA state matrix and the remaining action space, and then calculates the reward for this action. Here the reward is the sum of the differences between current state and native state. In addition, for the rendering of RNA secondary structure graphics, we use python’s network [35] extension package to visualize the final secondary structure by converting each base into a network node, and base pairing into a node connection.

## 3. Results

### 3.1. Learning the Fastest Folding Paths of Several Short RNAs

We selected several RNA molecules with a length of 30 nucleotides and used 2dRNA-Fold to learn and visualize their fastest folding paths. In the following we discuss these results separately.

**Figure 5:**
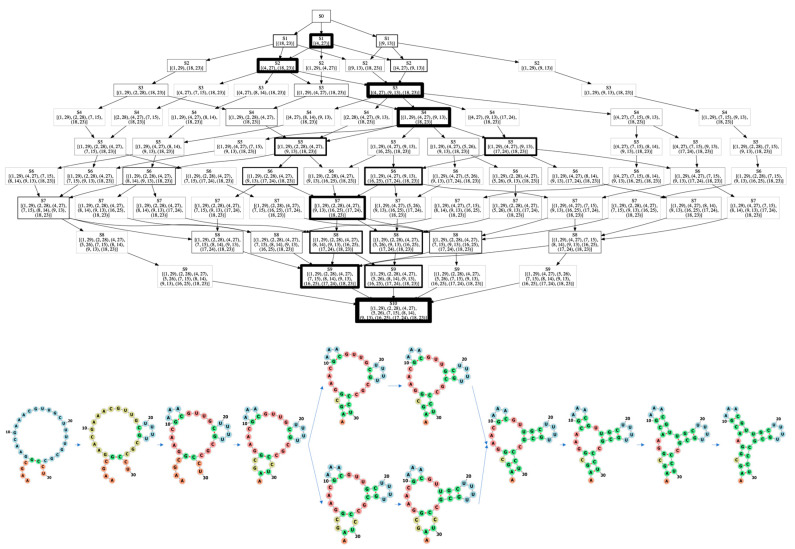
The learned fastest folding path of an RNA. The top is the folding path tree, each box represents a state, the thickness of the box represents the number of passes. The darker the color, the more times this state have been passed. The number in box is the indexes of the paired residues. And the bottom is the visualized folding path.

Figure 5 shows the learned fastest folding path of an RNA from [36]. Its sequence is AAGCGGAACGAAACGUUGCUUUUGCGCCCU and secondary structure is “.((.((.(((...)))(((....)))))))”, which has the native base pairs (2, 30), (3, 29), (5, 28), (6, 27), (8, 16), (9, 15), (10, 14), (17, 26), (18, 25), (19, 24), a bulge loop at the nucleotide 4 (C4) and a three-way loop. The upper part of Figure 5 shows the folding path tree obtained according to the path taken in each episode of the learning process, and the thickness of the box represents the number of passes. By counting the number of times that each node of the path tree has been traveled and connecting the largest one at each step, we get the final folding path, which is shown in the lower part of Figure 5. Here we assume that the most visited nodes in the folding path tree are the most probable intermediate states. At the beginning, the probability of selecting next node from the current node is the same since there is no information at this time to guide us to be biased towards a certain node. But after round after round of training, the learned value and policy network combined with scoring function in MCTS can assign remaining bases with different probabilities. From the folding path we can observe that this RNA molecule first forms the overall multi-loop structure through three key base-pairings, and then starts to generate two stems of the three-branched loop, and finally fills up and grows the last stem regions. For the two hairpins, the closest base pairs to the hairpin loops are first formed and then other base pairs are formed successively, i.e., a zipping way, which is in agreement with the observed folding mechanism of a hairpin molecule that the base pairing occurs via a small loop formation followed by zipping upon f-quench [20]. For the non-hairpin stem, the first formed base pair is not the closest one to the two ends.

**Figure 6:**
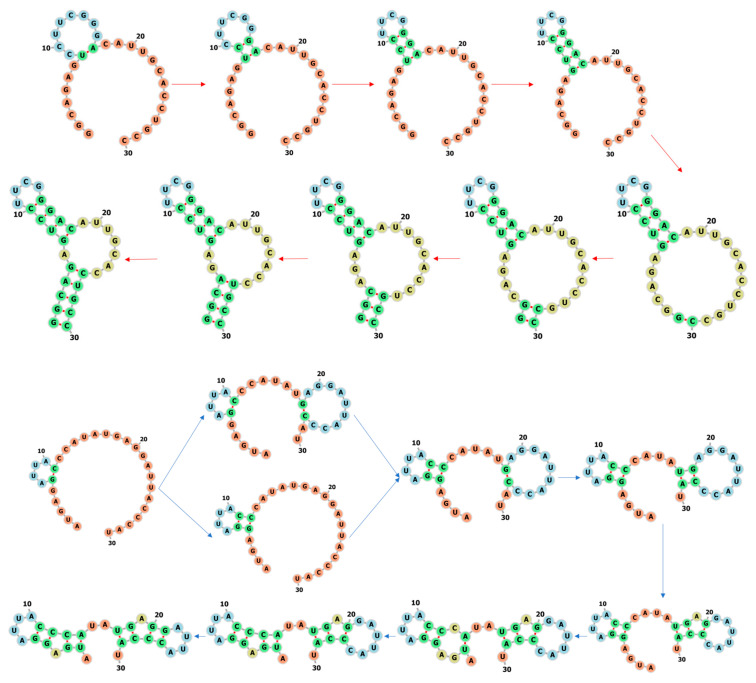
The prediction results of the fastest folding paths of two RNA molecules, PDB_00312 (**top**) and PDB_01136 (**bottom**).

Figure 6 shows the learned fastest folding paths of other two RNA molecules from CompaRNA [37]. One RNA with ID PDB_00312 has a sequence GGCAGAGUCCUUCGGGACAUUGCACCUGCC and secondary structure “(((((.((((....)))).......)))))”, which is a hairpin with an internal loop (two-way loop). The native base pairs are (1, 30), (2, 29), (3, 28), (4, 27), (5, 26), (7, 18), (8, 17), (9, 16), (10, 15). For this hairpin, the first formed base pair is the closest one to the hairpin loop and then other base pairs are formed successively to the internal loop. The stem with open end is formed from the base pair at the open end. Another RNA with ID PDB_01136 has a sequence AUGAGGAUUACCCAUAUGAGGAUUACCCAU and secondary structure “.((.((....))))..((.((....)))).”, which consists of two hairpins with bulges connected by a opened two-way loop. The native base pairs are (2, 14), (3, 13), (5, 12), (6, 11), (17, 29), (18, 28), (20, 27), (21, 26), and the two bulge loops are A4 and A19, respectively. From the folding path we also find that this RNA first forms the overall open two-way-loop structure through two key base-pairings, and then the two stems. This indeed is the fastest way to form the native secondary structure. The formation of the secondary structure also starts from the closest base pair to one of the hairpin loops.

### 3.2. Learning the Fastest Folding Path of a Riboswitch RNA

**Figure 7:**
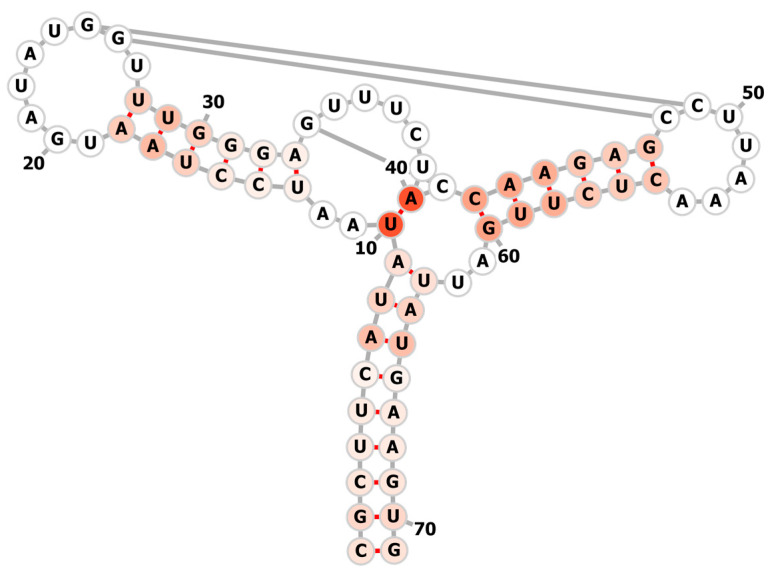
The learned fastest folding path of 1Y26. The darker the color, the earlier the base pairing will be formed.

We also selected a longer RNA molecule to test the capabilities of 2dRNA-Fold for learning the fastest folding path of longer RNAs. Here we chose one famous riboswitch RNA molecule with PDB ID 1Y26, the length is 71 nucleotides, its sequence is:

$$\begin{array}{l} \mathrm{CGCUUCAUAUAUAAUCCUAAUGAUAUGGUUUGGGAGUUUCUACCAAGA}\\ \mathrm{GCCUUAAACUCUUGAUUAUGAAGUG} \end{array}$$

and the secondary structure (Figure 7) is

“((((((((((..((((((......[[.))))))[.....)]((((((]].....))))))..)))))))))”.

Since the length of 1Y26 is relatively longer, the training time is very long. On average, each episode of folding process takes 4521.73 s, which is about 75.36 min. We trained a total of 960 episodes. In the folding path learning process, there is only one folding path that successfully folds to the native state, so we use the paths of near-native states (except for pseudoknots, the paths that the final folding state and the native state differ by one or two base pairs) to build a folding-path tree. Since the folding-path tree is very large with many nodes, we show the learned folding path more intuitively in Figure 7 in which each pair is scored in the order in which they appear in the folding path. The darker the color, the earlier the base pairing will be formed. It can be seen from the figure that the key position (10, 40) is formed at the beginning. Once this position is determined, the framework of the secondary structure of the entire RNA is basically determined. This is similar to the examples above. It can also be seen that the hairpin loop on the right is easily formed first, followed by the left-side hairpin, and the lower side last. The base pairing of the right hairpin loop is formed from the far end, this also has an experimental correspondence [20], which showed the T-quench refolding in hairpin formation has multiple pathways either starting by small loop or a large loop. Although our method does not explicitly consider the refolding mechanism, but our result possibly shows that it has learned this mechanism.

One thing worth noting here is that because 1Y26 contains three pseudoknots, our action space does not contain pseudoknots, but the native secondary structure we used in training has not been processed, that is, removing these three pseudoknots, 2dRNA-Fold cannot correctly predict out these three pseudoknots.

## 4. Discussion

### 4.1. The Impact of Monte Carlo Tree Search

**Figure 8:**
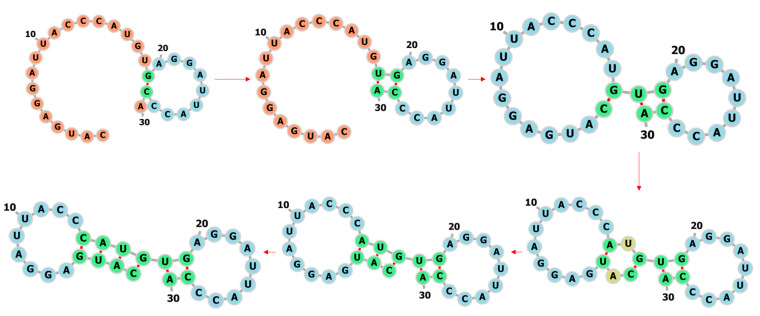
The fastest secondary structure folding path prediction results of PDB_00972.

We trained a model on the RNA molecule with ID PDB_00972. The RNA sequence is CAUGAGGGAUUACCCAUGUGAGGAUUACCCA, the length is 30, and the secondary structure is “(((((.........))))((.. .......))”, the native base pairs are (1, 17), (2, 16), (3, 15), (4, 14), (18, 30), (19, 29). The predicted fastest secondary structure folding path is shown in Figure 8.

**Figure 9:**
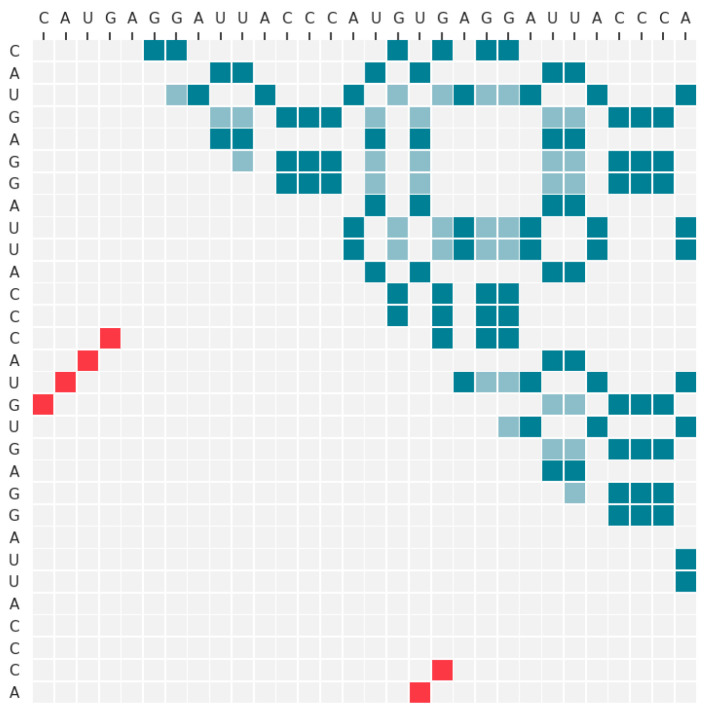
The secondary structure of RNA PDB_00972 and its initial action space. The native secondary structure is located in the lower right corner, indicated in red; the initial action space is located in the upper left half corner, indicated in blue.

Then we used this model to visualize the intermediate results in the step of selecting the first base pairing to reveal the effect of the Monte Carlo tree search. The secondary structure of the RNA and its initial action space are shown in Figure 9. It can be seen from the figure that the initial action space is very large, and the upper right corner is almost full. While there are only six native base pairs, it is quite difficult to select the correct pair from so many possible pairs.

**Figure 10:**
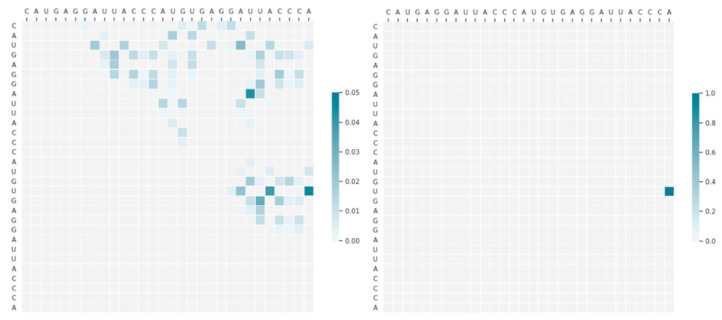
The action space probability matrix diagram of PDB_00972 (**left**) and the probability matrix diagram corrected by Monte Carlo tree search (**right**).

We input the initial state data into the 2dRNA-Fold model, through the policy network we can get a probability matrix of L×L, because not every entry in probability matrix can be paring, so we mask and select those probabilities that may be matched according to the action space, and normalize these values to 0 to 1 interval and then get the left image in Figure 10. From the figure, we can see that compared to the large number of possible choices in the original action space (Figure 9), the policy network has helped us filter out many possible base pairings, leaving only a few. Here, it can be clearly seen that there are three or four pairs with the darkest color (the highest probabilities of selection), and one of them is the native pair. However, the results of the policy network alone cannot identify the correct pairing from these three or four. At this time, a Monte Carlo tree search is required. The right image in Figure 10 is the corrected probability matrix after applying 2000 Monte Carlo tree searches. At this time, only one point with a probability close to 1 is left in the graph, and the other points are almost negligible because the probability is too small. The remaining base pairing at this position is exactly the native pairing. It shows that it is difficult to get the correct result only by the prediction result of the policy network, but with the assistance of Monte Carlo tree search, its accuracy will be greatly improved.

### 4.2. Multi-Threaded Accelerated Training and Its Impact

It takes a long time to train 2dRNA-Fold model. Compared with the time taken to train the neural network, a lot of time is mainly consumed in the process of Monte Carlo tree search.

However, in each episode and every step, we have to perform 2000 Monte Carlo tree searches. We tried to reduce the number of Monte Carlo tree searches. However, through experiments, we found that it would seriously affect the learning efficiency, so we adopted a multi-threaded approach to speed up training.

**Figure 11:**
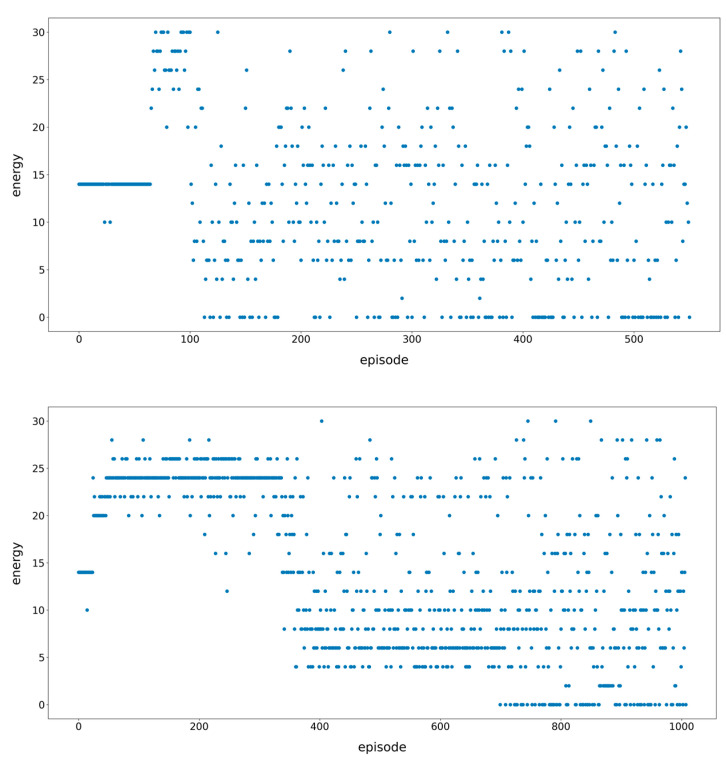
The scatter plot of training results with/without multi-threading accelerating. The picture **above** is the normal version, and the picture **below** is the multi-threaded acceleration version. In the figure, the y-axis is the error value between the final state and the native state after each episode, and the x-axis is the number of episodes.

We mainly perform multi-thread acceleration in the whole folding simulation part. Here, 20 threads are used, that is, 20 episodes of folding simulation are performed at the same time, but they share the same policy/value network parameters. When these 20 simulations are over, the generated data are put into the database, and then are sampled to train the neural network. After the neural network is trained, it continues to use 20 threads to perform folding simulation. As shown in Figure 11, we compared the results with and without multi-threading. When multi-threading is not used, the fluctuation is large. After more than 100 episodes, the correct folding path appears, and it is slowly stabilized after training to 500 episodes. At this time, the time consumed is 1939 min. After using multi-threading, the process of model training is steadily improved, and it is stabilized after about 1000 episodes. At this time, the time spent is only 352 min, which greatly shortens the training time.

### 4.3. Prediction of the Fastest Folding Path and Secondary Structure of RNA

**Figure 12:**
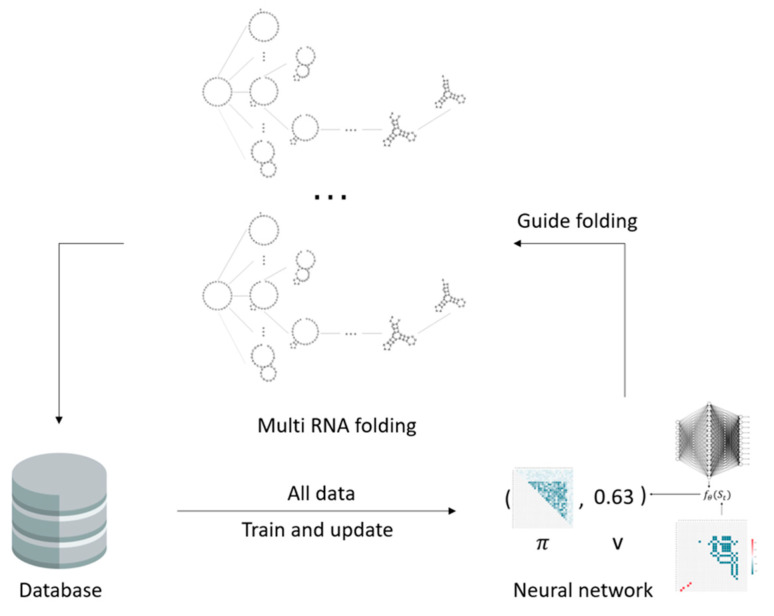
Diagram of multi-RNA molecular folding path learning.

2dRNA-Fold can be used to predict the fastest folding path and secondary structure of a new RNA at the same time when it is trained for a large number of RNA molecules by folding-path learning. The diagram of multi-RNA molecules folding path learning is shown in Figure 12.

As a first try, we used 770 RNA sequences less than 50 in the bpRNA [38] database as the training set, and the other 15 sequences as the test set. In each episode, we sampled each of the RNA sequences from the training set according to a certain probability to perform the folding of this episode, as shown below:

(5) $$P_{i}=\frac{e^{\frac{V_{i}}{N_{i}L_{i}}}}{\sum_{i}e^{\frac{V_{i}}{N_{i}L_{i}}}}$$

where $V_{i}$ represents the cumulative sum of errors between the final state and the native state of each ith RNA folding episode, the initial value is 1. $N_{i}$ represents the sampling times, and $L_{i}$ represents the length of the sequence. This formula effectively balances the sampling probability between the samples with poor prediction performance and the samples with very few sampling times.

**Figure 13:**
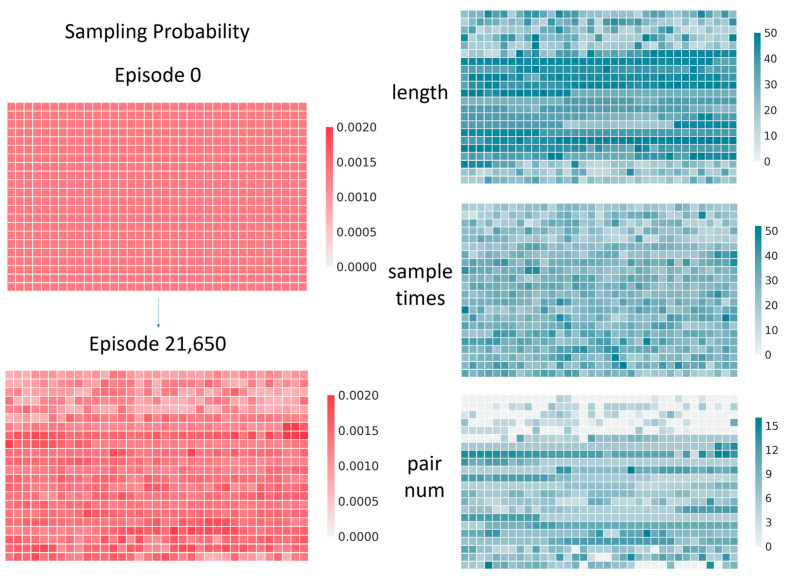
The sampling probability of each sample on the training set (**left**) and its length, samples times, and number of base pairs (**right**).

We performed 21,650 episodes of folding simulation on this training set and visualized the sampling probability of each sample on the training set, as shown in Figure 13. It can be seen that our sampling strategy is well balanced. It is not limited to more sampling of long sequences, but also increases the sampling probability for some samples with more base pairings.

**Figure 14:**
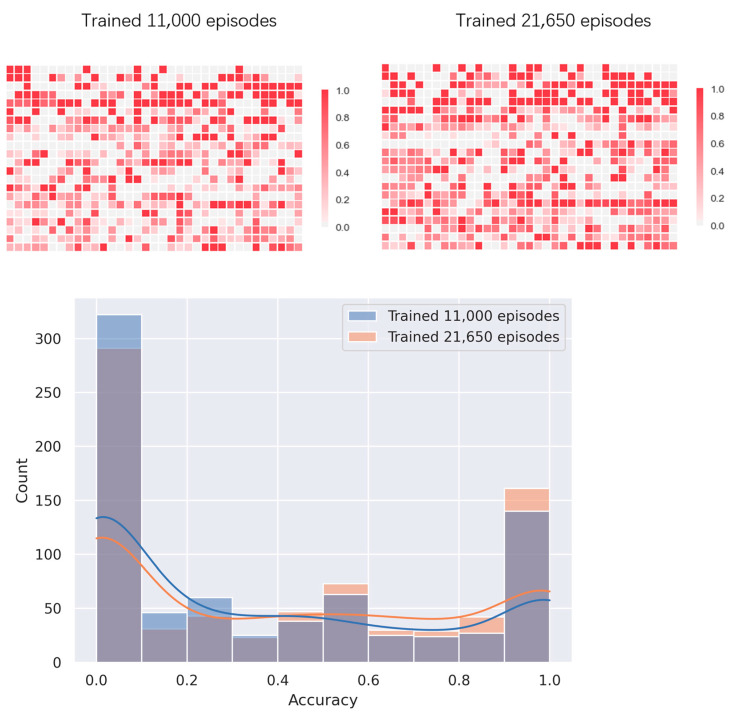
The prediction accuracy of each sample on the training set. On **the upper left** is the result at the 11,000th episode, and on **the upper right** is the result at the 21,650th episode. **The bottom** is the accuracy distribution chart.

Figure 14 shows the base pairing prediction accuracy of 2dRNA-Fold for each sample of the training set. We selected a point in the middle of training (training to 11,000th episode) as a comparison. At the 11,000th episode, the average accuracy of the model on the training set was 0.36 and rose to 0.42 after the 21,650 episodes. The accuracy on the test set has also been improved from 0.3 to 0.45. Although the accuracy of 2dRNA-Fold is not very high and even not as good as some traditional secondary structure prediction methods, it is mainly used to study the folding paths in the secondary structure formation, and it can be said to be an additional function to predict the secondary structure. Furthermore, another reason may be that our model is still under-fitting. It is obvious from Figure 14 that despite the improvement in 21,650 episodes of training, there are still many samples whose prediction results are still very poor, indicating that training is far from enough. Limited by computational capability, we do not have so many resources for a large amount of long-term training, so our model have not been further trained.

**Figure 15:**
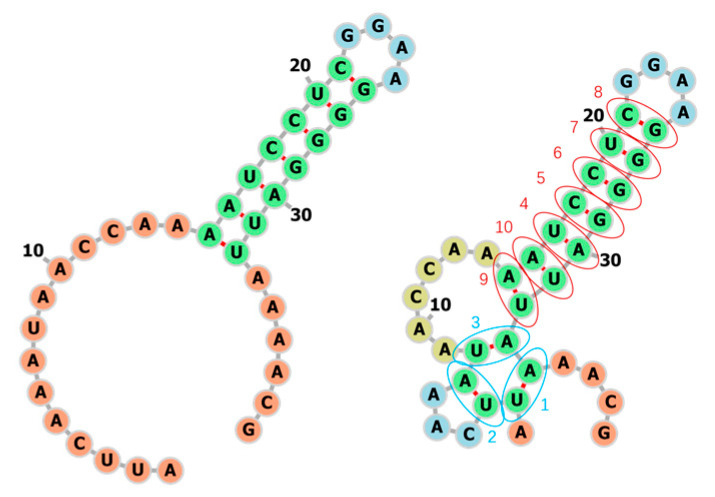
The predicted secondary structure and folding path of an RNA (bpRNA_RFAM_25409). The blue circle indicates the wrong prediction, the red indicates the correct prediction, and the numbers next to it indicate the order of formation (folding path).

We pick one RNA (bpRNA_RFAM_25409) from the test set to see if 2dRNA-Fold learned the rules from the multi-RNA training as same as that we used to learn with a single RNA before. The sequence of the RNA is AUUCAAAUAACCAAAAUCCUCGGAAGGGGAUUAAAACG, the length is 38 nucleotides, and the secondary structure is “..............(((((((....))))))))......”. The predicted fastest folding path is shown in Figure 15, with the native structure on the left and the predicted secondary structure on the right. Compared with the native structure, the prediction result has three more wrong pairs, but the stem part of the hairpin is correctly predicted. Of course, affected by the predicted three wrong pairings, the stem is formed beginning at the distal position from the hairpin loop. Another interesting thing is that any of the three A-U pairs can be formed first in six-membered ring or the stem of hairpin. The reason for this particular order we think maybe is that 2dRNA-Fold uses Monte Carlo tree search to select next base pair from a huge search space and the neural network will learn to find specific pairs to narrow the search space so that the native state can be reached more easily and quickly.

**Figure 16:**
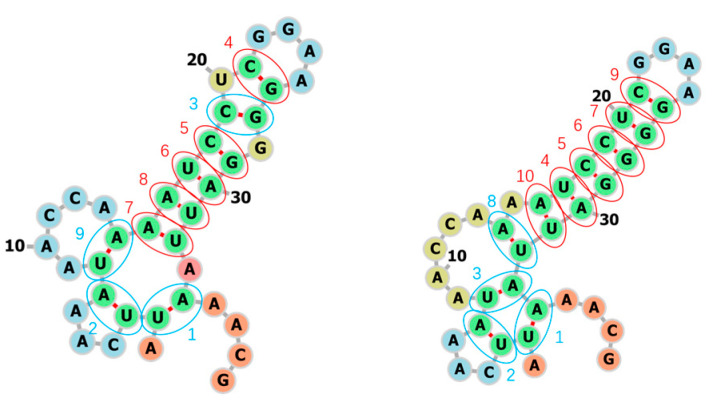
The other two predicted secondary structures and folding paths of bpRNA_RFAM_25409. The blue circle indicates the wrong prediction, the red indicates the correct prediction, and the numbers next to it indicate the order of base pair formation or folding paths.

What’s more, in the secondary structure prediction mode, 2dRNA-Fold takes nonstochastic sampling in the selection part of Monte Carlo tree search to avoid different prediction results each time and the predicted folding paths are the most likely folding paths or the fastest folding paths. If we do not remove this randomness, we can obtain multiple different potential folding pathways, but in this case these paths may be not the most likely folding paths or the fastest folding paths. The other two possible folding pathways for the above example (bpRNA_RFAM_25409) are shown in Figure 16.

Finally, it should be pointed out that in 2dRNA-Fold the scoring to determine next step is based on possible base pairings, the learned or predicted folding path may be not completely in agreement with the real folding paths that are determined by free energy according to physical laws. So, it may be better to incorporate free energy into the scoring function, so that the results obtained can be more consistent with the experimental studies. However, currently accurate calculation of free energy is difficult. But anyway 2dRNA-Fold can be easily extended to use scoring function based on free energy if there is an accurate method to calculate free energy of RNA secondary structure.

## 5. Conclusions

In summary, we developed a deep reinforcement learning algorithm called 2dRNA-Fold to learn the fastest folding paths in RNA secondary structure formation. 2dRNA-Fold uses a neural network combined with Monte Carlo tree search to select residue pairings. The learned fastest folding paths of some RNAs show that 2dRNA-Fold can quickly find the key base pairs that determine the framework of the native secondary structures. Furthermore, 2dRNA-Fold can also be used to predict RNA secondary structure if it is sufficiently trained on large dataset. Since our method does not combine physical mechanisms such as energy and thermodynamics, the identified fastest folding paths for RNAs may not be the actual folding paths.

## Data Availability

The trained model and some examples are available at SPOT-RNA and at https://github.com/Urinx/2dRNA-Fold (accessed on 20 July 2021).
